# Impact of vaccination and SARS-CoV-2 variants on severe COVID-19 outcomes: a cross-sectional study, Brazil, 2021-2022

**DOI:** 10.1590/S2237-96222025v34e20240613.en

**Published:** 2025-09-01

**Authors:** Luiza Paiva Moraes, Letícia Martins Raposo

**Affiliations:** 1 Universidade Federal do Estado do Rio de Janeiro, Centro de Ciências Exatas e Tecnologia, Rio de Janeiro, RJ, Brazil

**Keywords:** COVID-19 Vaccines, SARS-CoV-2, Respiration, Artificial, Mortality, Risk Factors, Vacunas contra la COVID-19, SARS-CoV-2, Respiración Artificial, Mortalidad, Factores de Riesgo

## Abstract

**Objective:**

To investigate association between vaccination, SARS-CoV-2 variants, and severe outcomes among Brazilian patients hospitalized due to COVID-19.

**Methods:**

This was a cross-sectional study using Brazilian Ministry of Health data on patients hospitalized with severe acute respiratory syndrome due to COVID-19 between 2021 and 2022. Demographic, clinical, and outcome variables were analyzed, considering SARS-CoV-2 variants and vaccination status. Statistical tests included Pearson’s chi-square test, Cochran-Armitage trend test, Mann-Whitney U test, Kruskal-Wallis test, and Poisson regression with robust variance to identify risk factors for invasive mechanical ventilation and death, estimating prevalence ratios (PR) and 95% confidence intervals (95%CI).

**Results:**

A total of 73,193 patients were analyzed. The Omicron variant was associated with lower rates of respiratory symptoms, invasive ventilation, and mortality compared to other variants. Vaccination significantly reduced the likelihood of invasive mechanical ventilation and death. Patients infected during the period of predominant Omicron circulation had 21.0% lower prevalence of invasive mechanical ventilation (PR 0.79; 95%CI 0.74; 0.84) and 25.0% lower prevalence of death (PR 0.75; 95%CI 0.72; 0.79). Full vaccination status also showed a protective effect, with a 22.0% reduction in prevalence of invasive mechanical ventilation (PR 0.78; 95%CI 0.72; 0.85) and a 16.0% reduction in prevalence of death (PR 0.84; 95%CI 0.79; 0.90), compared to unvaccinated individuals.

**Conclusion:**

The Omicron variant was associated with lower rates of invasive mechanical ventilation and mortality compared to previous variants, while vaccination demonstrated a significant protective effect by reducing severe outcomes.

## Resumen

**Objetivo:**

Investigar la asociación entre la vacunación, las variantes del SARS-CoV-2 y los resultados graves en brasileños hospitalizados por covid-19.

**Métodos:**

Se trata de un estudio transversal con datos del Ministerio de Salud sobre pacientes hospitalizados por síndrome respiratorio agudo grave debido a la covid-19 en Brasil entre 2021 y 2022. Se analizaron variables demográficas, clínicas y resultados, teniendo en cuenta las variantes del SARS-CoV-2 y el estado de vacunación. Las pruebas estadísticas incluyeron la prueba de chi cuadrado de Pearson, Cochran-Armitage, U de Mann-Whitney, Kruskal-Wallis y regresión de Poisson con varianza robusta para identificar factores de riesgo de ventilación mecánica invasiva y mortalidad, estimando la razón de prevalencia (RP) y el intervalo de confianza del 95% (IC95%).

**Resultados:**

Se analizaron 73 193 pacientes. La variante Ómicron presentó menores tasas de síntomas respiratorios, ventilación invasiva y mortalidad en comparación con otras variantes. La vacunación redujo significativamente las posibilidades de ventilación mecánica invasiva y muerte. Los pacientes infectados durante la mayor circulación de la variante Ómicron tuvieron un 21,0% menos de prevalencia de ventilación mecánica invasiva (RP 0,79; IC95% 0,74; 0,84) y un 25,0% menos de prevalencia de muerte (RP 0,75; IC95% 0,72; 0,79). La vacunación completa también mostró un efecto protector, con una reducción del 22,0% en la prevalencia de ventilación mecánica invasiva (RP 0,78; IC95% 0,72; 0,85) y del 16,0% en la prevalencia de mortalidad (RP 0,84; IC95% 0,79; 0,90) en comparación con los no vacunados.

**Conclusión:**

La variante Ómicron se asoció con menores tasas de ventilación invasiva y mortalidad en comparación con las variantes anteriores, mientras que la vacunación mostró un efecto protector significativo, reduciendo los resultados graves.

**Ethical aspects**

This research used public domain anonymized databases.

## Introduction

The COVID-19 pandemic has had a devastating impact on global public health, with nearly 7 million deaths reported by the World Health Organization (1). Brazil was severely affected, experiencing three waves of mortality that resulted in more than 700,000 confirmed deaths (2). Although the most critical phase of the pandemic has passed, retrospective analysis of vaccination strategies and the response to different SARS-CoV-2 variants remains relevant to reaffirm the importance of the interventions adopted.

The emergence of new SARS-CoV-2 variants, such as Gamma and Omicron, challenged the effectiveness of pandemic control measures. Variants with higher transmissibility overwhelmed healthcare systems and posed additional challenges to COVID-19 management (3,4). In Brazil, viral genetic sequencing identified the Gamma variant as predominant during the second wave of deaths, in March 2021 (5). This more transmissible variant than the original strain highlighted the need for a robust response, which included advancing vaccination campaigns.

Brazil began its vaccination campaign in January 2021, prioritizing high-risk groups such as healthcare workers, older adults, and individuals with comorbidities (6,7). The vaccines administered included CoronaVac (Sinovac Biotech), ChAdOx1 nCov-19 (AstraZeneca/University of Oxford), BNT162b2 (Pfizer-BioNTech), and Ad26.COV2.S (Johnson & Johnson–Janssen). Since then, vaccination has played a crucial role in reducing severe COVID-19 outcomes, even in the face of emerging variants.

With the emergence of the Omicron variant at the end of 2021, there was a significant increase in the number of cases, although with lower mortality compared to previous variants (5). As of January 2025, Brazil had administered more than 520 million doses of monovalent vaccines (6), reaching 86.39% of the population with two doses, 56.09% with three doses, and 19.48% with four doses (8). The introduction of bivalent vaccines reinforced the immune response against Omicron and its subvariants, with more than 36 million doses administered, emphasizing the importance of continued immunization in pandemic control (6). However, bivalent vaccine coverage remains low, reaching only 21.65% of the population (8).

Although several studies have examined vaccine effectiveness against severe outcomes, many have focused on specific populations, such as older adults or immunocompromised individuals (9-12), or addressed only specific variants at different points during the pandemic (13,14). Few studies have simultaneously assessed the impact of SARS-CoV-2 variants and vaccination on patients hospitalized for severe acute respiratory syndrome (SARS), especially in Brazil — one of the countries most affected by the pandemic. 

This study aimed to evaluate the impact of vaccination and SARS-CoV-2 variants on severe outcomes, such as the use of invasive mechanical ventilation and mortality, in patients hospitalized with SARS due to COVID-19 in Brazil. The analysis sought to confirm vaccine effectiveness and the impact of circulating variants on the clinical course of hospitalized patients, providing evidence to support future pandemic control and prevention strategies.

## Methods

### Study design

This was a cross-sectional analysis of secondary data on patients hospitalized with SARS due to COVID-19 in Brazil, from January 2021 to December 2022. 

### Setting

The data analyzed in this study covered all regions of Brazil and were extracted from the Ministry of Health’s SARS database, available on the openDataSUS platform. This database includes mandatory notifications of hospitalized SARS cases nationwide, covering both public and private healthcare facilities. SARS surveillance in Brazil was established by the Ministry of Health with effect from the influenza A(H1N1)pdm09 pandemic. In 2020, it was expanded to include COVID-19, thereby enabling comprehensive monitoring of the pandemic. The study period covered approximately two years of the COVID-19 vaccination campaign, from January 2021 to December 2022, a period marked by the gradual introduction of vaccines and the circulation of the Gamma, Delta, and Omicron variants.

### Participants

The study included patients aged 18 years or older who were hospitalized with confirmed diagnosis of SARS due to COVID-19 by RT-PCR testing of nasopharyngeal secretion samples collected within two days of admission. Only cases that resulted in cure or death due to COVID-19 were considered. 

Individuals lacking information on vaccination status or date of hospitalization were excluded, as were those with inconsistent data on the SARS-CoV-2 variant, including cases classified as “other” or involving co-circulation of multiple variants. Records with incomplete information on vaccination regimen, such as missing vaccination dates, manufacturer, or number of doses, were removed. It was also necessary to remove records with errors regarding the sequence of doses and records of patients without detailed data on intensive care unit (ICU) admission, invasive mechanical ventilation, signs and symptoms, comorbidities, and race/skin color (Supplementary Figure 1).

### Variables

The independent variables analyzed in this study were: sex (male, female); age (in years); race/skin color (White, Brown/Black, Asian/Indigenous); respiratory symptoms (dyspnea [yes, no], respiratory distress [yes, no], oxygen saturation <95% [yes, no]); comorbidities (chronic cardiovascular disease [yes, no], asthma [yes, no], diabetes [yes, no], obesity [yes, no]); SARS-CoV-2 variant (Gamma, Delta, Omicron); COVID-19 vaccination status (unvaccinated, incomplete vaccination regimen, primary vaccination regimen, full vaccination regimen); time from symptom onset to hospital admission (in days); length of hospitalization (in days); and ICU admission (yes, no).

The response variables analyzed in the study were use of invasive mechanical ventilation (yes, no) and death from COVID-19 (yes, no).

### Data sources and measurement

Data were obtained directly from the SARS database, with predominant variants identified using a proxy variable based on sequencing data from the GISAID Initiative and the Fiocruz Genomic Network (15). The dominant variant was assigned to each patient based on the date of symptom onset and the variant’s monthly prevalence. Only months in which the circulating variant reached a frequency of at least 70.0% were considered, ensuring that patients were predominantly exposed to a single variant. 

The temporal categorization of variants was as follows: Other variants (January 2021), Other variants and Gamma (February 2021), Gamma (March to July 2021), Gamma and Delta (August 2021), Delta (September to November 2021), Delta and Omicron (December 2021), Omicron (January to December 2022). Patients whose symptom onset occurred during periods of mixed variant circulation or in months classified as “other variants” were excluded.

Vaccination status was also extracted from the SARS database and classified into four categories: Unvaccinated (no record of vaccination), Incomplete vaccination regimen (one dose of CoronaVac, ChAdOx1 nCov-19, or BNT162b2), Primary vaccination regimen (two doses of CoronaVac, ChAdOx1 nCov-19, or BNT162b2, or one dose of Ad26.COV2.S), Full vaccination regimen (primary series plus booster dose). The distinction between partially vaccinated individuals and unvaccinated individuals has also been used in previous studies (16-18).

### Bias control

To reduce selection bias, individuals with missing or inconsistent data on vaccination, hospitalization, or clinical outcomes were excluded. Using data from a national database minimized sampling bias, and application of Poisson regression adjusted for multiple variables helped control for potential confounding factors.

### Statistical methods

This study compared demographic, clinical, and outcome variables in patients hospitalized with COVID-19 in Brazil, considering the predominance periods of the Delta, Gamma, and Omicron variants and vaccination status. Characteristics and outcomes of patients infected during the Omicron period were stratified by vaccination status. Categorical variables were described using absolute and relative frequencies, and numerical variables were presented as medians and interquartile ranges (IQR).

Statistical tests included: Pearson’s chi-square test for associations between categorical variables; Cochran-Armitage trend test for ordinal variables; Mann-Whitney U test for two-group comparisons; Kruskal-Wallis test for three or more groups, with Dunn’s post-hoc test and Bonferroni correction. Normality of numerical variables was assessed using the Kolmogorov-Smirnov test.

Poisson regression with robust variance was used to estimate risk factors for invasive mechanical ventilation and death, with results expressed as prevalence ratios (PR) and 95% confidence intervals (95%CI). Model fit was assessed using pseudo R^2^, indicating the proportion of explained variability. All analyses were conducted using R software, version 4.2.2 (R Foundation for Statistical Computing, Vienna, Austria).

### Data access and cleaning methods

The data used in this study are publicly available and were obtained from the Brazilian Ministry of Health. They were cleaned beforehand to remove duplicate entries and inconsistencies. Data quality checks were performed to ensure the integrity and consistency of key variables. Data processing and analysis were performed using R software, version 4.2.2. They are available at: https://github.com/leticiaraposo/Vacinacao_Variantes_covid19_Desfechos_Brasil_2021_2022 (19).

## Results

The characteristics of the 73,193 individuals hospitalized with SARS due to COVID-19 between 2021 and 2022 were presented, highlighting significant differences among the Delta, Gamma, and Omicron variants (Table 1). Male patients (52.6%) and those identifying as White (61.6%) predominated. Median age increased significantly over the analyzed periods, from 57 years (IQR 47-66) during the Gamma phase to 74 years (IQR 62-83) during the Omicron phase (p-value<0.001). Dyspnea, respiratory distress, and oxygen saturation <95% were more frequent in Delta and Gamma, with a decline during Omicron. Obesity was most prevalent during Gamma (22.4%) and least prevalent during Omicron (8.3%). Full vaccination status was more common during Omicron (46.4%). ICU admissions, use of mechanical ventilation, and mortality were lower during Omicron (34.2%). Time between symptom onset and hospital admission significantly decreased over the analyzed periods, with medians of 8 days (IQR 5-11) during Gamma, 6 days (IQR 4-9) during Delta, and 3 days (IQR 1-7) during Omicron (p-value <0.001). Hospitalization duration also varied: 8 days (IQR 5-15) during Gamma, 9 days (IQR 5-18) during Delta, and 7 days (IQR 4-14) during Omicron (p-value<0.001).

**Table 1:** Demographic, clinical, and outcome characteristics of individuals hospitalized with severe acute respiratory syndrome (SARS) due to COVID-19, according to the periods of predominance of SARS-CoV-2 variants. Brazil, 2021-2022 (n=73,193)

Variables	Total n=73,193 n (%)	Delta n=2,167 n (%)	Gamma n=56,233 n (%)	Omicron n=14,793 n (%)	p-value^a^
Sex					<0.001
Female	34,673 (47.4)	1,052 (48.5)	26,229 (46.6)	7,392 (50.0)	
Male	38,520 (52.6)	1,115 (51.5)	30,004 (53.4)	7,401 (50.0)	
**Race/skin color**					<0.001
Asian/Indigenous	792 (1.1)	40 (1.8)	566 (1.0)	186 (1.3)	
White	45,084 (61.6)	1,205 (55.6)	34,161 (60.7)	9,718 (65.7)	
Brown/Black	27,317 (37.3)	922 (42.5)	21,506 (38.2)	4,889 (33.0)	
Dyspnea					<0.001
No	16,278 (22.2)	513 (23.7)	11,023 (19.6)	4,742 (32.1)	
Yes	56,915 (77.8)	1,654 (76.3)	45,210 (80.4)	10,051 (67.9)	
**Respiratory distress**					<0.001
No	26,014 (35.5)	869 (40.1)	18,516 (32.9)	6,629 (44.8)	
Yes	47,179 (64.5)	1,298 (59.9)	37,717 (67.1)	8,164 (55.2)	
**Oxygen saturation** <95%					<0.001
No	16,469 (22.5)	565 (26.1)	10,961 (19.5)	4,943 (33.4)	
Yes	56,724 (77.5)	1,602 (73.9)	45,272 (80.5)	9,850 (66.6)	
**Chronic cardiovascular disease**					<0.001
No	34,528 (47.2)	1,010 (46.6)	26,965 (48.0)	6,553 (44.3)	
Yes	38,665 (52.8)	1,157 (53.4)	29,268 (52.0)	8,240 (55.7)	
Asthma					0.042
No	70,106 (95.8)	2,060 (95.1)	53,833 (95.7)	14,213 (96.1)	
Yes	3,087 (4.2)	107 (4.9)	2,400 (4.3)	580 (3.9)	
Diabetes					0.617
No	48,236 (65.9)	1,443 (66.6)	37,011 (65.8)	9,782 (66.1)	
Yes	24,957 (34.1)	724 (33.4)	19,222 (34.2)	5,011 (33.9)	
Obesity					<0.001
No	58,972 (80.6)	1,785 (82.4)	43,619 (77.6)	13,568 (91.7)	
Yes	14,221 (19.4)	382 (17.6)	12,614 (22.4)	1,225 (8.3)	
**COVID-19 vaccination**					<0.001
Unvaccinated	51,785 (70.8)	1,244 (57.4)	47,328 (84.2)	3,213 (21.7)	
Partially vaccinated	9,686 (13.2)	515 (23.8)	8,416 (15.0)	755 (5.1)	
Primary	4,851 (6.6)	400 (18.5)	489 (0.9)	3,962 (26.8)	
Fully vaccinated	6,871 (9.4)	8 (0.4)	0 (0.0)	6,863 (46.4)	
**Admission to intensive care unit**					<0.001
No	42,562 (58.2)	1,053 (48.6)	32,466 (57.7)	9,043 (61.1)	
Yes	30,631 (41.8)	1,114 (51.4)	23,767 (42.3)	5,750 (38.9)	
**Use of invasive mechanical ventilation**					<0.001
No	53,617 (73.3)	1,570 (72.5)	40.006 (71.1)	12,041 (81.4)	
Yes	19,576 (26.7)	597 (27.5)	16,227 (28.9)	2,752 (18.6)	
**Death from COVID**-19					<0.001
No	43,480 (59.4)	1,297 (59.9)	32,445 (57.7)	9,738 (65.8)	
Yes	29,713 (40.6)	870 (40.1)	23,788 (42.3)	5,055 (34.2)	

^a^Pearson’s chi-square test.

Differences were observed between vaccinated (at least one dose) and unvaccinated individuals hospitalized during the Delta and Omicron periods (Table 2). During Delta, no significant differences were found for most variables, except for lower frequency of oxygen saturation <95% among vaccinated individuals (p-value 0.044). During Omicron, vaccinated individuals showed lower frequency of dyspnea (p-value 0.001), respiratory distress (p-value 0.001), and use of mechanical ventilation (p-value<0.001), with no significant difference in mortality (p-value 0.064). 

**Table 2:** Characteristics and outcomes of vaccinated and unvaccinated individuals hospitalized with COVID-19 during the periods of predominance of the Delta and Omicron variants. Brazil, 2021-2022 (n=16,960)

Variables	Total n (%)	Unvaccinated n (%)	Vaccinated (at least 1 dose) n (%)	p-value^a^
Delta period	n=2,167	n=1,244	n=923	
Sex				0.119
Female	1,052 (48.5)	586 (47.1)	466 (50.5)	
Male	1,115 (51.5)	658 (52.9)	457 (49.5)	
**Race/skin color**				0.110
Asian/Indigenous	1,205 (55.6)	669 (53.8)	536 (58.1)	
White	922 (42.5)	549 (44.1)	373 (40.4)	
Brown/Black	40 (1.8)	26 (2.1)	14 (1.5)	
Dyspnea				0.385
No	513 (23.7)	286 (23.0)	227 (24.6)	
Yes	1,654 (76.3)	958 (77.0)	696 (75.4)	
**Respiratory distress**				0.188
No	869 (40.1)	484 (38.9)	385 (41.7)	
Yes	1,298 (59.9)	760 (61.1)	538 (58.3)	
**Oxygen saturation** <95%				0.044
No	565 (26.1)	304 (24.4)	261 (28.3)	
Yes	1,602 (73.9)	940 (75.6)	662 (71.7)	
**Admission to intensive care unit**				0.278
No	1,053 (48.6)	592 (47.6)	461 (49.9)	
Yes	1,114 (51.4)	652 (52.4)	462 (50.1)	
**Use of invasive mechanical ventilation**				0.901
No	1,570 (72.5)	900 (72.3)	670 (72.6)	
Yes	597 (27.5)	344 (27.7)	253 (27.4)	
**Death from COVID**-19				0.355
No	1,297 (59.9)	755 (60.7)	542 (58.7)	
Yes	870 (40.1)	489 (39.3)	381 (41.3)	
Omicron period	n=14,793	n=3,213	n=11,580	
Sex				0.014
Female	7,392 (50.0)	1,544 (48.1)	5,848 (50.5)	
Male	7,401 (50.0)	1,669 (51.9)	5,732 (49.5)	
**Race/skin color**				<0.001
Asian/Indigenous	9,718 (65.7)	1,822 (56.7)	7,896 (68.2)	
White	4,889 (33.0)	1,349 (42.0)	3,540 (30.6)	
Brown/Black	186 (1.3)	42 (1.3)	144 (1.2)	
Dyspnea				0.001
No	4,742 (32.1)	955 (29.7)	3,787 (32.7)	
Yes	10,051 (67.9)	2,258 (70.3)	7,793 (67.3)	
**Respiratory distress**				0.001
No	6,629 (44.8)	1,360 (42.3)	5,269 (45.5)	
Yes	8,164 (55.2)	1,853 (57.7)	6,311 (54.5)	
**Oxygen saturation** <95%				0.361
No	4,943 (33.4)	1,052 (32.7)	3,891 (33.6)	
Yes	9,850 (66.6)	2,161 (67.3)	7,689 (66.4)	
**Admission to intensive care unit**				0.087
No	9,043 (61.1)	2,006 (62.4)	7,037 (60.8)	
Yes	5,750 (38.9)	1,207 (37.6)	4,543 (39.2)	
**Use of invasive mechanical ventilation**				<0.001
No	12,041 (81.4)	2,545 (79.2)	9,496 (82.0)	
Yes	2,752 (18.6)	668 (20.8)	2,084 (18.0)	
**Death from COVID**-19				0.064
No	9,738 (65.8)	2,071 (64.5)	7,667 (66.2)	
Yes	5,055 (34.2)	1,142 (35.5)	3,913 (33.8)	

^a^Pearson’s chi-square test.

Individuals with a full vaccination regimen presented lower frequencies of dyspnea (p-value <0.001), respiratory distress (p-value 0.005), use of mechanical ventilation (p-value<0.001), and COVID-19-related death (p-value 0.001) (Table 3). Median age was significantly higher among those with complete vaccination regimen (77 years, IQR 68-85) compared to other vaccination groups (p-value<0.001).

**Table 3:** Characteristics and outcomes of vaccinated and unvaccinated individuals hospitalized vwith COVID-19 during the period of predominance of the Omicron variant. Brazil, 2021-2022 (n=14,793)

Variables	Unvaccinated n (%) n=3,213	Incomplete vaccination regimen n (%) n=755	Primary vaccination regimen n (%) n=3,962	Full vaccination regimen n (%) n=6,863	p-value
Sex					0.012^a^
Female	1,544 (48.1)	359 (47.5)	1,972 (49.8)	3,517 (51.2)	
Male	1,669 (51.9)	396 (52.5)	1,990 (50.2)	3,346 (48.8)	
**Race/skin color**					<0.001^a^
Asian/Indigenous	1,822 (56.7)	430 (57.0)	2,516 (63.5)	4,950 (72.1)	
White	1,349 (42.0)	316 (41.9)	1,410 (35.6)	1,814 (26.4)	
Brown/Black	42 (1.3)	9 (1.2)	36 (0.9)	99 (1.4)	
Dyspnea					<0.001^b^
No	955 (29.7)	227 (30.1)	1,295 (32.7)	2,265 (33.0)	
Yes	2,258 (70.3)	528 (69.9)	2,667 (67.3)	4,598 (67.0)	
**Respiratory distress**					0.005^b^
No	1,360 (42.3)	337 (44.6)	1,817 (45.9)	3,115 (45.4)	
Yes	1,853 (57.7)	418 (55.4)	2,145 (54.1)	3,748 (54.6)	
**Oxygen saturation** <95%					0.879^b^
No	1,052 (32.7)	260 (34.4)	1,363 (34.4)	2,268 (33.0)	
Yes	2,161 (67.3)	495 (65.6)	2,599 (65.6)	4,595 (67.0)	
**Admission to intensive care unit**					0.857^b^
No	2,006 (62.4)	433 (57.4)	2,366 (59.7)	4,238 (61.8)	
Yes	1,207 (37.6)	322 (42.6)	1,596 (40.3)	2,625 (38.2)	
**Use of invasive mechanical ventilation**					<0.001^b^
No	2,545 (79.2)	599 (79.3)	3,163 (79.8)	5,734 (83.5)	
Yes	668 (20.8)	156 (20.7)	799 (20.2)	1,129 (16.5)	
**Death from COVID**-19					0.001^b^
No	2,071 (64.5)	483 (64.0)	2,547 (64.3)	4,637 (67.6)	
Yes	1,142 (35.5)	272 (36.0)	1,415 (35.7)	2,226 (32.4)	

^a^Pearson’s chi-square test; ^b^Cochran-Armitage trend test.

Adjusted prevalence rates for invasive mechanical ventilation and death in individuals hospitalized for COVID-19 were presented, with emphasis on associated factors (Table 4). The model adjusted for mechanical ventilation showed 6.80% pseudo R^2^, while the model adjusted for death showed 25.36% pseudo R^2^. Male patients had higher risk of mechanical ventilation (PR 1.07; 95%CI 1.05; 1.10) and death (PR 1.07; 95%CI 1.05; 1.09) compared to females. Older age was associated with increased risk of death (PR 1.02; 95%CI 1.02; 1.02). Individuals with oxygen saturation <95% had greater risks of mechanical ventilation (PR 1.41; 95%CI 1.36; 1.47) and death (PR 1.14; 95%CI 1.11; 1.17). 

**Table 4:** Adjusted prevalence ratio (PR) and 95% confidence interval (95%CI) for invasive mechanical ventilation and death, according to the study variables. Brazil, 2021-2022 (n=73,193)

	Invasive mechanical ventilation		Death	
Variables	PR (95%IC)	p-value	PR (95%IC)	p-value
Sex				
Female	1.00		1.00	
Male	1.07 (1.05; 1.10)	<0.001	1.07 (1.05; 1.09)	<0.001
**Age** (years)	1.00 (1.00; 1.01)	<0.001	1.02 (1.02; 1.02)	<0.001
**Race/skin color**				
White	1.00		1.00	
Brown/Black	1.03 (1.01; 1.06)	0.012	1.04 (1.02; 1.06)	<0.001
Asian/Indigenous	1.04 (0.92; 1.16)	0.556	0.92 (0.85; 0.99)	0.031
Dyspnea				
No	1.00		1.00	
Yes	1.30 (1.25; 1.35)	<0.001	1.10 (1.07; 1.12)	<0.001
**Respiratory distress**				
No	1.00		1.00	
Yes	1.33 (1.29; 1.38)	<0.001	1.10 (1.08; 1.12)	<0.001
**Oxygen saturation** <95%				
No	1.00		1.00	
Yes	1.41 (1.36; 1.47)	<0.001	1.14 (1.11; 1.17)	<0.001
**Chronic cardiovascular disease**				
No	1.00		1.00	
Yes	1.07 (1.04; 1.10)	<0.001	0.97 (0.95; 0.98)	<0.001
Asthma				
No	1.00		1.00	
Yes	0.94 (0.88; 1.00)	0.044	0.93 (0.90; 0.97)	0.001
Diabetes				
No	1.00		1.00	
Yes	1.14 (1.11; 1.17)	<0.001	1.05 (1.04; 1.07)	<0.001
Obesity				
No	1.00		1.00	
Yes	1.42 (1.38; 1.46)	<0.001	1.06 (1.04; 1.08)	<0.001
**Use of invasive mechanical ventilation**				
No			1.00	
Yes			2.15 (2.11; 2.20)	<0.001
**Admission to intensive care unit**				
No			1.00	
Yes			1.77 (1.73; 1.81)	<0.001
**Time from symptom onset to hospital admission** (days)	1.00 (1.00; 1.00)	0.016	0.98 (0.98; 0.99)	<0.001
**Length of hospital stay** (days)	1.01 (1.01; 1.01)	<0.001	1.00 (1.00; 1.00)	<0.001
**SARS-CoV-2 variants**				
Gamma	1.00		1.00	
Delta	0.98 (0.91; 1.05)	0.541	0.82 (0.78; 0.85)	<0.001
Omicron	0.79 (0.75; 0.84)	<0.001	0.75 (0.73; 0.78)	<0.001
**COVID-19 vaccination**				
Unvaccinated	1.00		1.00	
Partially vaccinated	0.95 (0.92; 0.99)	0.005	0.99 (0.97; 1.01)	0.201
Primary series	0.98 (0.92; 1.05)	0.541	1.00 (0.96; 1.04)	0.917
Fully vaccinated	0.78 (0.73; 0.85)	<0.001	0.84 (0.81; 0.88)	<0.001

Those infected with the Omicron variant had lower risks of mechanical ventilation (PR 0.79; 95%CI 0.75; 0.84) and death (PR 0.75; 95%CI 0.73; 0.78) compared to Gamma. Full vaccination status reduced the risk of mechanical ventilation (PR 0.78; 95%CI 0.73; 0.85) and death (PR 0.84; 95%CI 0.81; 0.88) compared to unvaccinated individuals. Use of mechanical ventilation was associated with death (PR 2.15; 95%CI 2.11; 2.20), as was ICU admission (PR 1.77; 95%CI 1.73; 1.81).

## Discussion

This study identified significant differences among the Gamma, Delta, and Omicron variants in terms of demographic profile, clinical manifestations, comorbidities, and outcomes. The Omicron variant was associated with less severe clinical disease, which was seen in lower rates of invasive mechanical ventilation and mortality. It could be observed that full vaccination status offered substantial protection against severe outcomes, with a more pronounced impact during the period of Omicron predominance. Older age, male sex, and comorbidities such as obesity were identified as important risk factors for adverse outcomes, reinforcing the vulnerability of these groups and the need for effective preventive strategies such as vaccination.

Distribution of patients between the three variants varied significantly, with a progressive increase in the median age of patients over the periods. The highest rate was observed during the Omicron period. These findings are consistent with data from the main university medical center in Ljubljana, Slovenia, between 2021 and 2022, which showed predominance of older patients during the Omicron wave (20). In contrast, younger populations were also observed during this period in different geographic and institutional settings (16,21). Higher median age of patients infected during the Omicron wave has been linked to greater effectiveness of vaccination among younger individuals (22,23). Moreover, in Slovenia, the lower severity of Omicron was marked by hospitalization of older and frailer patients even in less severe cases, unlike the Delta wave, which was marked by greater severity and hospital demand (20).

When analyzing clinical symptoms across the three periods, lower prevalence of severe symptoms — such as dyspnea and low oxygen saturation — was observed during the Omicron wave. This finding is consistent with data from a large community healthcare network in the United States, which assessed 9,582 hospitalized patients with COVID-19 between March 2020 and February 2022 during the pre-Delta, Delta, and Omicron periods (24). They also reported lower frequencies of hypoxemia among vaccinated individuals during the Delta and Omicron periods.

Full vaccination status was associated with reduced respiratory distress, fever, and dyspnea in patients with COVID-19-related acute respiratory distress syndrome, as observed in a cohort of 265 intubated patients in Greece between June 2021 and February 2022. During that period, the Delta variant predominated, and fully vaccinated patients showed lower clinical severity and reduced likelihood of death (25). Our study also confirmed that full complete vaccination status reduces the risk of mortality and the need for invasive mechanical ventilation, even among patients with comorbidities, emphasizing its role in mitigating COVID-19 severity.

In our study, hospitalization duration during the Omicron period was longer (median of one week) compared to previous findings reporting shorter stays (26,27). This difference may be explained by the inclusion of more severe SARS cases in this analysis, although the findings are consistent with the aforementioned United States setting (24). During Omicron, hospitalization was significantly shorter for patients who had received more vaccine doses, which emphasized the cumulative effect of vaccination. In Norway, between February and November 2021, shorter hospital stays and lower ICU admission risk were also observed among vaccinated patients, although ICU was not separately assessed in that study (28). Omicron appears to be associated with faster progression of upper respiratory tract symptoms, which may explain the shorter time between symptom onset and hospitalization compared to Delta (29,30).

The Omicron period showed the lowest rates of severe outcomes, such as invasive mechanical ventilation and death — results that were corroborated by previous studies. (16,24,26,27,31). During the Omicron wave, observed mainly between November and December 2021, various regions reported significant reductions in the clinical severity of COVID-19. In South Africa, among 971 hospitalized patients, lower oxygen dependency, mechanical ventilation use, and mortality were recorded compared to previous waves (26). At the Johns Hopkins health system in the United States, data on 2,027 patients indicated lower ICU admissions, mechanical ventilation use, and mortality compared to the Delta variant (16). In Paris, between November 29, 2021, and January 10, 2022, an analysis of 1,716 emergency department visits also showed reduced ICU admissions, mechanical ventilation use, and mortality (27). Between July 2021 and January 2022, at a teaching hospital in Los Angeles, California, similarly lower rates of mechanical ventilation and mortality were observed during the Omicron period, consistent with the findings of this study (31).

Poisson regression revealed that advanced age, male sex, and Brown/Black race/skin color variables increased the prevalence of mechanical ventilation and death. These results are consistent with data obtained in Brazil between February 2021 and January 2022, which also identified age as a risk factor, especially among hospitalized vaccinated individuals (32). In a sample of 72,647 patients hospitalized with severe COVID-19, men had higher risk of mortality, corroborating the findings of the present study. Vaccination was again highlighted as a protective factor, since it significantly reduced the risk of mechanical ventilation and death. In Brazil, administration of booster doses increased the effectiveness of vaccines in preventing severe cases and deaths, with a sustained protective effect for several weeks after administration (33). However, in Norway, data from hospitalized patients between February and November 2021 (28) suggested that vaccination may not substantially alter mortality risk among very elderly individuals, especially those over 80 years of age.

These findings underscore the importance of continuous immunization strategies to mitigate severe COVID-19 outcomes, particularly in older and high-risk populations. The progressive reduction in vaccine protection over time, attributed to immunosenescence and a lower immune response (34,35), makes it essential to administer booster doses, which temporarily maintain high efficacy (36-38). Introducing booster doses during Omicron predominance may have contributed to the reduced case severity observed, emphasizing the need for updated vaccination policies to extend immunity and protect vulnerable populations.

This study was notable for its large sample size, which gave it high statistical power. Furthermore, the careful selection of statistical analyses ensured the robustness of the results. As with any large-scale study, sample losses were expected but were minimized by applying rigorous inclusion and exclusion criteria, ensuring data quality and reducing potential confounding.

Some limitations should be noted. Variant identification was indirect, based on temporal predominance, which may lead to inaccuracies, particularly in regions with limited genomic surveillance. The lack of detailed data on vaccine type, number of doses, and precise time since vaccination limited more specific analyses of vaccination regimens. Including only hospitalized patients restricted the generalizability of the findings, and factors such as coinfections, differences in care quality, and regional variations may have influenced outcomes. Additionally, selection bias should be considered, as the sample represented only a portion of SARS cases during the analyzed period.

Although the large sample size strengthened the findings, small differences may not be clinically relevant. Given that this was a cross-sectional study, only associations could be identified, and not causal relationships. Longitudinal studies are needed for a more in-depth assessment of the impacts of vaccination and variant circulation on COVID-19 clinical outcomes.

The findings of this study underscore the dynamic nature of the COVID-19 pandemic and the importance of ongoing surveillance of SARS-CoV-2 variants to guide public health strategies. The lower clinical severity associated with the Omicron variant, combined with the protective impact of vaccination on severe outcomes, reinforces the need for high vaccination coverage and adaptation of immunization campaigns in response to emerging variants. Identification of risk factors such as older age, male sex, and comorbidities highlights the importance of targeted preventive approaches for vulnerable groups. These results provided fundamental evidence for the formulation of COVID-19 mitigation policies. In addition, they highlighted the essential role of vaccination and epidemiological surveillance in containing the impacts of the pandemic.

## Data Availability

The database and analysis codes used in the research are available from: https://github.com/leticiaraposo/Vacinacao_Variantes_covid19_Desfechos_Brasil_2021_2022.

## Supplementary material

Supplementary table 1.
