# Supplementary material for: Impact of vaccination and SARS-CoV-2 variants on severe COVID-19 outcomes: a cross-sectional study, Brazil, 2021-2022
Source: Epidemiol Serv Saude. 2025 Sep 1;34:e20240613. doi: 10.1590/S2237-96222025v34e20240613.en (PMC12404605; doi:10.1590/S2237-96222025v34e20240613.en)
Supplement: Supplementary file 1 [file 2237-9622-ress-34-e20240613-suppl01-en.pdf]

Figura suplementar 1. Processo de seleção da amostra do estudo, incluindo critérios de inclusão e exclusão. Brasil, 2021-2022 (n=73.193)

Initial database
Total = 2,268,112 records
North (5.6%)
Northeast (18.2%)
Midwest (8.7%)
Southeast (49.4%)
South (18.1%)

↓

Eligible:
Aged 18 years or older
Hospitalized with SARS due to COVID-19
Positive RT-PCR
Nasopharyngeal sample
Recovery or death due to COVID-19
Total = 601,371 records

↓

Exclusion:
"Other" variants or mixed variants
Total = 459,286 records

↓

Exclusion:
No vaccination data
Total = 317,864 records

↓

Exclusion:
Records with invalid doses
Total = 308,772 records

↓

Exclusion:
Vaccination dates outside 2021–2022
Total = 308,661 records

↓

Exclusion:
Inconsistencies in number of doses and vaccine manufacturers
Total = 257,149 records

↓

Exclusion:
Pregnant/postpartum individuals and people with Down syndrome
Total = 250,965 records

↓

Filtering:
Complete records (no missing data)
Final total = 73,193 records
North (1.7%)
Northeast (9.0%)
Midwest (9.4%)
Southeast (57.4%)
South (22.5%)
