# Supplementary material for: Impact of vaccination and SARS-CoV-2 variants on severe COVID-19 outcomes: a cross-sectional study, Brazil, 2021-2022
Source: Epidemiol Serv Saude. 2025 Sep 1;34:e20240613. doi: 10.1590/S2237-96222025v34e20240613.en (PMC12404605; doi:10.1590/S2237-96222025v34e20240613.en)

Figura suplementar 1. Processo de seleção da amostra do estudo, incluindo critérios de inclusão e exclusão. Brasil, 2021-2022 (n=73.193)

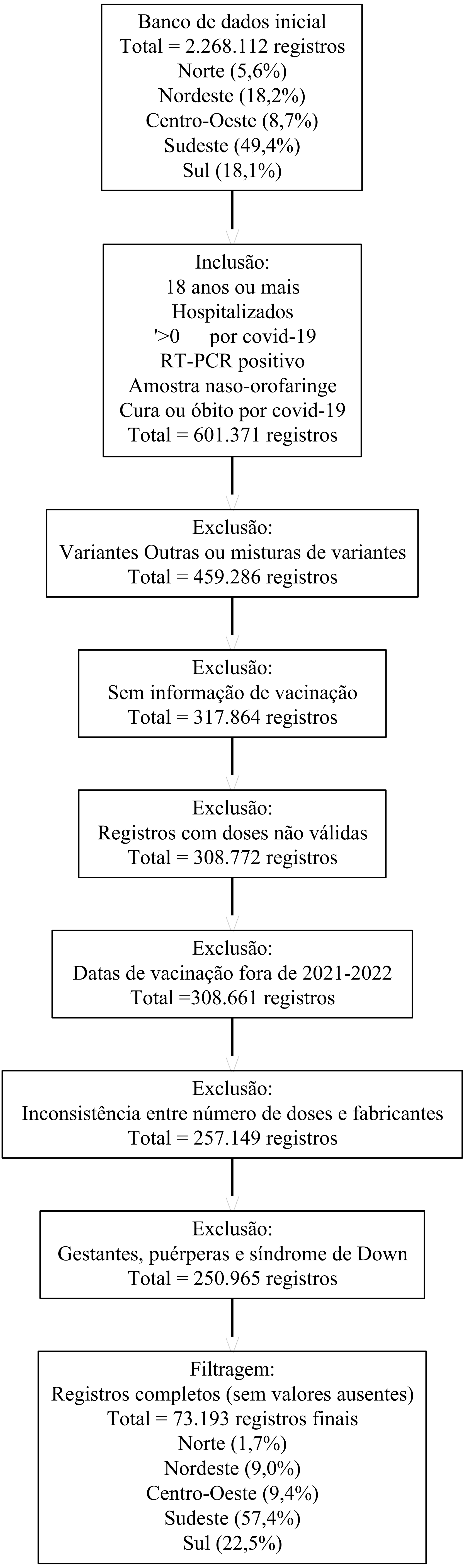

Supplement: Supplementary file 2 [file 2237-9622-ress-34-e20240613-suppl01-pt.pdf]
