# Supplementary material for: Impact of vaccination and SARS-CoV-2 variants on severe COVID-19 outcomes: a cross-sectional study, Brazil, 2021-2022
Source: Epidemiol Serv Saude. 2025 Sep 1;34:e20240613. doi: 10.1590/S2237-96222025v34e20240613.en (PMC12404605; doi:10.1590/S2237-96222025v34e20240613.en)
Supplement: Translation

# Translation: Impacto da vacinação e variantes do SARS-CoV-2 nos desfechos graves da covid-19: estudo transversal, Brasil, 2021-2022

**DOI:** 10.1590/S2237-96222025v34e20240613.pt

**Authors:** Luiza Paiva Moraes, Letícia Martins Raposo

**Affiliations:** 1 Universidade Federal do Estado do Rio de Janeiro, Centro de Ciências Exatas e Tecnologia, Rio de Janeiro, RJ, Brasil

**Keywords:** Vacinas Contra Covid-19, SARS-CoV-2, Respiração Artificial, Mortalidade, Fatores de Risco

## Abstract

**Objetivo:**

Investigar a associação entre vacinação, variantes do SARS-CoV-2 e desfechos graves em brasileiros hospitalizados por covid-19.

**Métodos:**

Tratou-se de estudo transversal com dados do Ministério da Saúde sobre pacientes hospitalizados por síndrome respiratória aguda grave devido à covid-19 no Brasil entre 2021 e 2022. Analisaram-se variáveis demográficas, clínicas e desfechos, considerando variantes do SARS-CoV-2 e status vacinal. Testes estatísticos incluíram qui-quadrado de Pearson, Cochran-Armitage, U de Mann-Whitney, Kruskal-Wallis e regressão de Poisson com variância robusta para identificar fatores de risco para ventilação mecânica invasiva e mortalidade, estimando razão de prevalência (RP) e intervalo de confiança de 95% (IC95%).

**Resultados:**

Foram analisados 73.193 pacientes. A variante Ômicron apresentou menores taxas de sintomas respiratórios, ventilação invasiva e mortalidade em comparação com outras variantes. A vacinação reduziu significativamente as chances de ventilação mecânica invasiva e óbito. Pacientes infectados durante a maior circulação da variante Ômicron tiveram 21,0% menos prevalência de ventilação mecânica invasiva (RP 0,79; IC95% 0,74; 0,84) e 25,0% menos prevalência de óbito (RP 0,75; IC95% 0,72; 0,79). A vacinação completa também apresentou efeito protetor, com redução de 22,0% na prevalência de ventilação mecânica invasiva (RP 0,78; IC95% 0,72; 0,85) e 16,0% na prevalência de óbito (RP 0,84; IC95% 0,79; 0,90) em comparação aos não vacinados.

**Conclusão:**

A variante Ômicron foi associada a menores taxas de ventilação invasiva e mortalidade em comparação com as variantes anteriores, enquanto a vacinação mostrou efeito protetor significativo, reduzindo desfechos graves.

**Aspectos éticos**

Esta pesquisa utilizou bancos de dados de domínio público e anonimizados.

## Introdução

A pandemia de covid-19 teve impacto devastador na saúde pública global, com quase 7 milhões de mortes relatadas pela Organização Mundial da Saúde (1). O Brasil foi severamente afetado, visto que enfrentou três ondas de mortalidade que resultaram em mais de 700 mil óbitos confirmados (2). Embora a fase mais crítica da pandemia tenha sido superada, a análise retrospectiva das estratégias de vacinação e da resposta às diferentes variantes do SARS-CoV-2 continua sendo relevante para a confirmação da importância das intervenções adotadas.

O surgimento de novas variantes do SARS-CoV-2, como Gama e Ômicron, desafiou a eficácia das medidas de controle da pandemia. Variantes com maior transmissibilidade sobrecarregaram os sistemas de saúde e impuseram obstáculos adicionais ao gerenciamento da covid-19 (3,4). No Brasil, o sequenciamento genético do vírus permitiu identificar a variante Gama como predominante durante a segunda onda de óbitos, em março de 2021 (5) conforme semana epidemiológica (SE. Essa variante, mais transmissível que a cepa original, destacou a necessidade de resposta robusta, o que incluiu o avanço das campanhas de vacinação.

O Brasil iniciou a campanha de vacinação em janeiro de 2021 e priorizou grupos de risco, como profissionais de saúde, idosos e indivíduos com comorbidades (6,7). As vacinas administradas incluíram CoronaVac (Sinovac Biotech), ChAdOx1 nCov-19 (AstraZeneca/Universidade de Oxford), BNT162b2 (Pfizer-BioNTech) e Ad26.Cov2.S (Johnson & Johnson-Janssen). A partir de então, a vacinação desempenhou papel crucial na redução dos desfechos graves da covid-19, mesmo diante do surgimento de novas variantes.

Com a emergência da variante Ômicron no final de 2021, houve aumento significativo no número de casos, embora com menor mortalidade em relação às variantes anteriores (5) conforme semana epidemiológica (SE). Até janeiro de 2025, o Brasil já havia administrado mais de 520 milhões de doses de vacinas monovalentes (6), alcançando 86,39% da população com duas doses, 56,09% com três doses e 19,48% com quatro doses (8). A introdução das vacinas bivalentes reforçou a resposta imunológica contra a Ômicron e suas subvariantes, com mais de 36 milhões de doses aplicadas, e evidenciou a importância da imunização contínua no controle da pandemia (6). No entanto, a cobertura vacinal com a bivalente permanece baixa, visto que atinge apenas 21,65% da população (8).

Embora diversos estudos tenham analisado a eficácia das vacinas contra desfechos graves, muitos focaram em populações específicas, como idosos ou imunossuprimidos (9-12), ou abordaram apenas variantes específicas em diferentes momentos da pandemia (13,14). Ainda são limitados os estudos que avaliam simultaneamente o impacto das variantes do SARS-CoV-2 e da vacinação em pacientes hospitalizados por síndrome respiratória aguda grave (SRAG), especialmente no Brasil, um dos países mais afetados pela pandemia. 

Este estudo teve como objetivo avaliar o impacto da vacinação e das variantes do SARS-CoV-2 nos desfechos graves, como o uso de ventilação mecânica invasiva e a mortalidade, em pacientes hospitalizados com SRAG associada à covid-19 no Brasil. A análise visou confirmar a eficácia das vacinas e o impacto das variantes na evolução clínica dos pacientes, oferecendo subsídios para futuras estratégias de controle e prevenção de pandemias.

## Métodos

### Desenho do estudo

Tratou-se de análise de estudo transversal de dados secundários de pacientes hospitalizados com SRAG causada pela covid-19, coletados no Brasil entre janeiro de 2021 e dezembro de 2022. 

### Contexto

Os dados analisados neste estudo abrangeram todas as regiões do Brasil e foram extraídos do banco de SRAG do Ministério da Saúde, disponível na plataforma openDataSUS. Esse banco reúne notificações obrigatórias de casos hospitalizados de SRAG em todo o país e abrange unidades de saúde públicas e privadas. A vigilância de SRAG no Brasil foi instituída pelo Ministério da Saúde desde a pandemia de influenza A(H1N1)pdm09 e, em 2020, passou a incluir a covid-19, permitindo amplo monitoramento da pandemia. O período do estudo abrangeu cerca de dois anos de campanha de vacinação contra a covid-19, de janeiro de 2021 a dezembro de 2022, período marcado pela introdução gradual das vacinas e pela circulação das variantes Gama, Delta e Ômicron.

### Participantes

Foram incluídos no estudo pacientes com 18 anos ou mais, hospitalizados com diagnóstico de SRAG por covid-19, confirmado pelo exame RT-PCR de amostras de secreção de nasofaringe, coletadas até dois dias após a hospitalização. Consideraram-se apenas os casos de cura ou óbito por covid-19. 

Foram excluídos indivíduos sem informações sobre status vacinal ou data de hospitalização, bem como aqueles com dados inconsistentes sobre a variante do SARS-CoV-2, incluindo casos classificados como “outras” ou de circulação simultânea de múltiplas variantes. Foram removidos registros com informações incompletas sobre o regime vacinal, como ausência de data de vacinação, fabricante ou número de doses. Também foram removidos registros com erros sobre a sequência de doses e registros de pacientes sem dados detalhados sobre unidade de terapia intensiva (UTI), ventilação mecânica invasiva, sinais e sintomas, comorbidades e raça/cor da pele (Figura suplementar 1).

### Variáveis

As variáveis independentes analisadas neste estudo foram: sexo (masculino, feminino); idade (em anos); raça/cor da pele (branca, parda/preta, amarela/indígena); sintomas respiratórios (dispneia [sim, não], desconforto respiratório [sim, não], saturação de O_2_<95% [sim, não]); comorbidades (doença cardiovascular crônica [sim, não], asma [sim, não] , diabetes [sim, não], obesidade [sim, não]); variante do SARS-CoV-2 (Gama, Delta, Ômicron); vacinação contra a covid-19 (não vacinado, esquema incompleto, esquema primário, esquema completo); tempo entre o início dos sintomas e a admissão hospitalar (em dias); duração da hospitalização (em dias); e admissão em UTI (sim, não).

As variáveis respostas analisadas no estudo foram uso de ventilação mecânica invasiva (sim, não) e óbito por covid-19 (sim, não).

### Fontes de dados e mensuração

Os dados foram obtidos diretamente da base de SRAG, sendo as variantes predominantes identificadas por meio de variável proxy, utilizando informações de sequenciamento da GISAID Initiative e da Rede de Genômica da Fiocruz (15). A variante dominante foi atribuída a cada paciente com base na data dos primeiros sintomas e na prevalência da variante durante o mês de infecção. Foram considerados apenas os casos em que a variante circulante atingiu a frequência de 70,0% ou mais no mês, garantindo que os pacientes estivessem predominantemente expostos a uma única variante nesse período. 

A categorização temporal das variantes seguiu os seguintes critérios: outras variantes (janeiro de 2021), outras variantes e Gama (fevereiro de 2021), Gama (março a julho de 2021), Gama e Delta (agosto de 2021), Delta (setembro a novembro de 2021), Delta e Ômicron (dezembro de 2021) e Ômicron (janeiro a dezembro de 2022). Foram excluídos da análise os pacientes cujo início dos sintomas ocorreu em períodos em que a variante predominante foi classificada como “outras” ou períodos de mistura significativa de variantes.

O status vacinal dos pacientes também foi extraído da base SRAG, sendo classificados em quatro categorias: não vacinados (sem registro de vacinação), esquema vacinal incompleto (uma dose de CoronaVac, ChAdOx1 nCov-19 ou BNT162b2), esquema vacinal primário (duas doses de CoronaVac, ChAdOx1 nCov-19 ou BNT162b2, ou uma dose de Ad26.Cov2.S) e esquema vacinal completo (esquema primário mais dose de reforço). A distinção entre indivíduos com esquema vacinal incompleto, ou seja, parcialmente vacinados, e aqueles não vacinados também foi utilizada em estudos anteriores (16-18).

### Controle de viés

Foram excluídos da análise indivíduos com informações ausentes ou inconsistentes sobre vacinação, internação ou desfechos clínicos, visando reduzir o viés de seleção. O uso de dados de um banco nacional minimizou o viés de amostragem, e o emprego de regressão de Poisson ajustada para múltiplas variáveis ajudou a controlar possíveis fatores de confusão.

### Métodos estatísticos

O estudo comparou variáveis demográficas, clínicas e desfechos de pacientes hospitalizados com covid-19 no Brasil, considerando os períodos de predominância das variantes Delta, Gama e Ômicron e o status vacinal. Foram analisadas características e desfechos em pacientes infectados durante a circulação da variante Ômicron, estratificados por status vacinal. Variáveis categóricas foram descritas por frequências absolutas e porcentagens, e numéricas, como mediana e intervalo interquartil (IIQ).

Os testes estatísticos incluíram: qui-quadrado de Pearson, para associações entre variáveis categóricas; Cochran-Armitage, para tendências ordinais; U de Mann-Whitney, para comparações entre dois grupos; Kruskal-Wallis, para três ou mais grupos; e post-hoc de Dunn com correção de Bonferroni. A normalidade das variáveis numéricas foi verificada pelo teste de Kolmogorov-Smirnov.

A regressão de Poisson com variância robusta estimou fatores de risco para ventilação mecânica e mortalidade, com razão de prevalência (RP) e intervalo de confiança de 95% (IC95%). A adequação dos modelos foi avaliada pelo pseudo R^2^, indicando a proporção da variabilidade explicada. As análises foram realizadas no software R versão 4.2.2 (R Foundation for Statistical Computing, Viena, Áustria).

### Acesso aos dados e métodos de limpeza

Os dados utilizados são de acesso público, disponibilizados pelo Ministério da Saúde, e foram previamente limpos para eliminar duplicatas e inconsistências. Realizaram-se verificações de qualidade para garantir a integridade e a consistência das variáveis de interesse. Os dados foram tratados e analisados no software R versão 4.2.2 e estão disponíveis em: https://github.com/leticiaraposo/Vacinacao_Variantes_covid19_Desfechos_Brasil_2021_2022 (19).

## Resultados

Apresentaram-se as características dos 73.193 indivíduos hospitalizados com SRAG por covid-19 entre 2021-2022, destacando diferenças significativas entre as variantes Delta, Gama e Ômicron (Tabela 1). Pacientes do sexo masculino (52,6%) e brancos (61,6%) predominaram. A mediana da idade aumentou significativamente ao longo dos períodos analisados, passando de 57 anos (IIQ 47-66), na fase da variante Gama, para 74 anos (IIQ 62-83), na fase da variante Ômicron (p-valor<0,001). Dispneia, desconforto respiratório e saturação de O₂<95% foram mais frequentes com Delta e Gama, reduzindo-se na Ômicron. Obesidade foi mais prevalente na Gama (22,4%) e menor na Ômicron (8,3%). A vacinação completa foi mais comum na Ômicron (46,4%). Admissões em UTI, ventilação mecânica e óbitos foram menores na Ômicron (34,2%). O tempo entre o início dos sintomas e a admissão hospitalar apresentou redução significativa ao longo dos períodos analisados, com medianas de 8 dias (IIQ 5–11) na fase da variante Gama, 6 dias (IIQ 4-9) na fase da variante Delta e 3 dias (IIQ 1-7) na fase da variante Ômicron (p-valor<0,001). A duração da hospitalização também variou entre os períodos, sendo de 8 dias (IIQ 5-15) na fase Gama, 9 dias (IIQ 5-18) na fase Delta e 7 dias (IIQ 4-14) na fase Ômicron (p-valor<0,001).

**Tabela 1:** Características demográficas, clínicas e de desfecho dos indivíduos hospitalizados com síndrome respiratória aguda grave (SRAG) devido à covid-19, segundo os períodos de predominância das variantes do SARS-CoV-2. Brasil, 2021-2022 (n=73.193)

Variáveis	Total n=73.193 n (%)	Delta n=2.167 n (%)	Gama n=56.233 n (%)	Ômicron n=14.793 n (%)	p-valor^a^
Sexo					<0,001
Feminino	34.673 (47,4)	1.052 (48,5)	26.229 (46,6)	7.392 (50,0)	
Masculino	38.520 (52,6)	1.115 (51,5)	30.004 (53,4)	7.401 (50,0)	
**Raça/cor da pele**					<0,001
Amarela/indígena	792 (1,1)	40 (1,8)	566 (1,0)	186 (1,3)	
Branca	45.084 (61,6)	1.205 (55,6)	34.161 (60,7)	9.718 (65,7)	
Parda/preta	27.317 (37,3)	922 (42,5)	21.506 (38,2)	4.889 (33,0)	
Dispneia					<0,001
Não	16.278 (22,2)	513 (23,7)	11.023 (19,6)	4.742 (32,1)	
Sim	56.915 (77,8)	1.654 (76,3)	45.210 (80,4)	10.051 (67,9)	
Desconforto respiratório					<0,001
Não	26.014 (35,5)	869 (40,1)	18.516 (32,9)	6.629 (44,8)	
Sim	47.179 (64,5)	1.298 (59,9)	37.717 (67,1)	8.164 (55,2)	
**Saturação de O**_2_<95%					<0,001
Não	16.469 (22,5)	565 (26,1)	10.961 (19,5)	4.943 (33,4)	
Sim	56.724 (77,5)	1.602 (73,9)	45.272 (80,5)	9.850 (66,6)	
**Doença cardiovascular crônica**					<0,001
Não	34.528 (47,2)	1.010 (46,6)	26.965 (48,0)	6.553 (44,3)	
Sim	38.665 (52,8)	1.157 (53,4)	29.268 (52,0)	8.240 (55,7)	
Asma					0,042
Não	70.106 (95,8)	2.060 (95,1)	53.833 (95,7)	14.213 (96,1)	
Sim	3.087 (4,2)	107 (4,9)	2.400 (4,3)	580 (3,9)	
Diabetes					0,617
Não	48.236 (65,9)	1.443 (66,6)	37.011 (65,8)	9.782 (66,1)	
Sim	24.957 (34,1)	724 (33,4)	19.222 (34,2)	5.011 (33,9)	
Obesidade					<0,001
Não	58.972 (80,6)	1.785 (82,4)	43.619 (77,6)	13.568 (91,7)	
Sim	14.221 (19,4)	382 (17,6)	12.614 (22,4)	1.225 (8,3)	
**Vacinação contra a covid**-19					<0,001
Não vacinado	51.785 (70,8)	1.244 (57,4)	47.328 (84,2)	3.213 (21,7)	
Incompleta	9.686 (13,2)	515 (23,8)	8.416 (15,0)	755 (5,1)	
Primária	4.851 (6,6)	400 (18,5)	489 (0,9)	3.962 (26,8)	
Completa	6.871 (9,4)	8 (0,4)	0 (0,0)	6.863 (46,4)	
**Admissão em unidade de terapia intensiva**					<0,001
Não	42.562 (58,2)	1.053 (48,6)	32.466 (57,7)	9.043 (61,1)	
Sim	30.631 (41,8)	1.114 (51,4)	23.767 (42,3)	5.750 (38,9)	
**Uso de ventilação mecânica invasiva**					<0,001
Não	53.617 (73,3)	1.570 (72,5)	40.006 (71,1)	12.041 (81,4)	
Sim	19.576 (26,7)	597 (27,5)	16.227 (28,9)	2.752 (18,6)	
**Óbito por covid**-19					<0,001
Não	43.480 (59,4)	1.297 (59,9)	32.445 (57,7)	9.738 (65,8)	
Sim	29.713 (40,6)	870 (40,1)	23.788 (42,3)	5.055 (34,2)	

^a^Teste qui-quadrado de Pearson.

Houve diferenças entre vacinados (pelo menos uma dose) e não vacinados hospitalizados com covid-19 durante as variantes Delta e Ômicron (Tabela 2). No período da Delta, não houve diferenças significativas entre os grupos para a maioria das variáveis, exceto menor frequência de saturação de O₂<95% em vacinados (p-valor 0,044). No período da Ômicron, vacinados apresentaram menor frequência de dispneia (p-valor 0,001), desconforto respiratório (p-valor 0,001) e uso de ventilação mecânica (p-valor<0,001), sem diferença significativa nos óbitos (p-valor 0,064). 

**Tabela 2:** Características e desfechos de indivíduos hospitalizados, vacinados e não vacinados contra a covid-19, durante os períodos de predominância das variantes Delta e Ômicron. Brasil, 2021-2022 (n=16.960)

Variáveis	Total n (%)	Não vacinado n (%)	Vacinado (pelo menos 1 dose) n (%)	p-valor^a^
Período da Delta	n=2.167	n=1.244	n=923	
Sexo				0,119
Feminino	1.052 (48,5)	586 (47,1)	466 (50,5)	
Masculino	1.115 (51,5)	658 (52,9)	457 (49,5)	
**Raça/cor da pele**				0,110
Amarela/indígena	1.205 (55,6)	669 (53,8)	536 (58,1)	
Branca	922 (42,5)	549 (44,1)	373 (40,4)	
Parda/preta	40 (1,8)	26 (2,1)	14 (1,5)	
Dispneia				0,385
Não	513 (23,7)	286 (23,0)	227 (24,6)	
Sim	1.654 (76,3)	958 (77,0)	696 (75,4)	
**Desconforto respiratório**				0,188
Não	869 (40,1)	484 (38,9)	385 (41,7)	
Sim	1.298 (59,9)	760 (61,1)	538 (58,3)	
**Saturação de O**_2_<95%				0,044
Não	565 (26,1)	304 (24,4)	261 (28,3)	
Sim	1.602 (73,9)	940 (75,6)	662 (71,7)	
**Admissão em unidade de terapia intensiva**				0,278
Não	1.053 (48,6)	592 (47,6)	461 (49,9)	
Sim	1.114 (51,4)	652 (52,4)	462 (50,1)	
**Uso de ventilação mecânica invasiva**				0,901
Não	1.570 (72,5)	900 (72,3)	670 (72,6)	
Sim	597 (27,5)	344 (27,7)	253 (27,4)	
**Óbito por covid**-19				0,355
Não	1.297 (59,9)	755 (60,7)	542 (58,7)	
Sim	870 (40,1)	489 (39,3)	381 (41,3)	
Período da Ômicron	n=14.793	n=3.213	n=11.580	
Sexo				0,014
Feminino	7.392 (50,0)	1.544 (48,1)	5.848 (50,5)	
Masculino	7.401 (50,0)	1.669 (51,9)	5.732 (49,5)	
**Raça/cor da pele**				<0,001
Amarela/indígena	9.718 (65,7)	1.822 (56,7)	7.896 (68,2)	
Branca	4.889 (33,0)	1.349 (42,0)	3.540 (30,6)	
Parda/preta	186 (1,3)	42 (1,3)	144 (1,2)	
Dispneia				0,001
Não	4.742 (32,1)	955 (29,7)	3.787 (32,7)	
Sim	10.051 (67,9)	2.258 (70,3)	7.793 (67,3)	
**Desconforto respiratório**				0,001
Não	6.629 (44,8)	1.360 (42,3)	5.269 (45,5)	
Sim	8.164 (55,2)	1.853 (57,7)	6.311 (54,5)	
**Saturação de O**_2_<95%				0,361
Não	4.943 (33,4)	1.052 (32,7)	3.891 (33,6)	
Sim	9.850 (66,6)	2.161 (67,3)	7.689 (66,4)	
**Admissão em unidade de terapia intensiva**				0,087
Não	9.043 (61,1)	2.006 (62,4)	7.037 (60,8)	
Sim	5.750 (38,9)	1.207 (37,6)	4.543 (39,2)	
**Uso de ventilação mecânica invasiva**				<0,001
Não	12.041 (81,4)	2.545 (79,2)	9.496 (82,0)	
Sim	2.752 (18,6)	668 (20,8)	2.084 (18,0)	
**Óbito por covid**-19				0,064
Não	9.738 (65,8)	2.071 (64,5)	7.667 (66,2)	
Sim	5.055 (34,2)	1.142 (35,5)	3.913 (33,8)	

^a^Teste qui-quadrado de Pearson.

Indivíduos com esquema vacinal completo apresentaram menor frequência de dispneia (p-valor<0,001), desconforto respiratório (p-valor 0,005), uso de ventilação mecânica (p-valor<0,001) e óbitos por covid-19 (p-valor 0,001) (Tabela 3). A mediana de idade foi significativamente maior entre os indivíduos com vacinação completa (77 anos, IIQ 68-85 anos) em comparação aos demais grupos de vacinação (p-valor<0,001).

**Tabela 3:** Características e desfechos de indivíduos hospitalizados, vacinados e não vacinados contra a covid-19, durante o período de predominância da variante Ômicron. Brasil, 2021-2022 (n=14.793)

Variáveis	Não vacinado n (%) n=3.213	Esquema incompleto n (%) n=755	Esquema primário n (%) n=3.962	Esquema completo n (%) n=6.863	p-valor
Sexo					0,012^a^
Feminino	1.544 (48,1)	359 (47,5)	1.972 (49,8)	3.517 (51,2)	
Masculino	1.669 (51,9)	396 (52,5)	1.990 (50,2)	3.346 (48,8)	
**Raça/cor da pele**					<0,001^a^
Amarela/indígena	1.822 (56,7)	430 (57,0)	2.516 (63,5)	4.950 (72,1)	
Branca	1.349 (42,0)	316 (41,9)	1.410 (35,6)	1.814 (26,4)	
Parda/preta	42 (1,3)	9 (1,2)	36 (0,9)	99 (1,4)	
Dispneia					<0,001^b^
Não	955 (29,7)	227 (30,1)	1.295 (32,7)	2.265 (33,0)	
Sim	2.258 (70,3)	528 (69,9)	2.667 (67,3)	4.598 (67,0)	
**Desconforto respiratório**					0,005^b^
Não	1.360 (42,3)	337 (44,6)	1.817 (45,9)	3.115 (45,4)	
Sim	1.853 (57,7)	418 (55,4)	2.145 (54,1)	3.748 (54,6)	
**Saturação de O**_2_<95%					0,879^b^
Não	1.052 (32,7)	260 (34,4)	1.363 (34,4)	2.268 (33,0)	
Sim	2.161 (67,3)	495 (65,6)	2.599 (65,6)	4.595 (67,0)	
**Admissão em unidade de terapia intensiva**					0,857^b^
Não	2.006 (62,4)	433 (57,4)	2.366 (59,7)	4.238 (61,8)	
Sim	1.207 (37,6)	322 (42,6)	1.596 (40,3)	2.625 (38,2)	
**Uso de ventilação mecânica invasiva**					<0,001^b^
Não	2.545 (79,2)	599 (79,3)	3.163 (79,8)	5.734 (83,5)	
Sim	668 (20,8)	156 (20,7)	799 (20,2)	1.129 (16,5)	
**Óbito por covid**-19					0,001^b^
Não	2.071 (64,5)	483 (64,0)	2.547 (64,3)	4.637 (67,6)	
Sim	1.142 (35,5)	272 (36,0)	1.415 (35,7)	2.226 (32,4)	

^a^Teste qui-quadrado de Pearson; ^b^Teste de Cochran-Armitage

Apresentaram-se razões de prevalência ajustadas para ventilação mecânica invasiva e óbito em indivíduos hospitalizados por covid-19, com destaque para fatores associados (Tabela 4). O modelo ajustado para ventilação mecânica invasiva apresentou pseudo R^2^ de 6,80%, enquanto o modelo para óbito apresentou pseudo R^2^ de 25,36%. Homens apresentaram maiores riscos de ventilação mecânica (RP 1,07; IC95% 1,05; 1,10) e óbito (RP 1,07; IC95% 1,05; 1,09) em comparação às mulheres. A idade avançada foi associada ao aumento no risco de óbito (RP 1,02; IC95% 1,02; 1,02). Indivíduos com saturação de O₂<95% apresentaram maiores riscos de ventilação mecânica (RP 1,41; IC95% 1,36; 1,47) e óbito (RP 1,14; IC95% 1,11; 1,17). 

**Tabela 4:** Razão de prevalência (RP) e intervalo de confiança de 95% (IC95%) ajustados para ventilação mecânica invasiva e óbito, segundo as variáveis do estudo. Brasil, 2021-2022 (n=73.193)

	Ventilação mecânica invasiva		Óbito	
Variáveis	RP (IC95%)	p-valor	RP (IC95%)	p-valor
Sexo				
Feminino	1,00		1,00	
Masculino	1,07 (1,05; 1,10)	<0,001	1,07 (1,05; 1,09)	<0,001
**Idade** (anos)	1,00 (1,00; 1,01)	<0,001	1,02 (1,02; 1,02)	<0,001
**Raça/cor da pele**				
Branca	1,00		1,00	
Parda/preta	1,03 (1,01; 1,06)	0,012	1,04 (1,02; 1,06)	<0,001
Amarela/indígena	1,04 (0,92; 1,16)	0,556	0,92 (0,85; 0,99)	0,031
Dispneia				
Não	1,00		1,00	
Sim	1,30 (1,25; 1,35)	<0,001	1,10 (1,07; 1,12)	<0,001
**Desconforto respiratório**				
Não	1,00		1,00	
Sim	1,33 (1,29; 1,38)	<0,001	1,10 (1,08; 1,12)	<0,001
**Saturação de O**_2_<95%				
Não	1,00		1,00	
Sim	1,41 (1,36; 1,47)	<0,001	1,14 (1,11; 1,17)	<0,001
**Doença cardiovascular crônica**				
Não	1,00		1,00	
Sim	1,07 (1,04; 1,10)	<0,001	0,97 (0,95; 0,98)	<0,001
Asma				
Não	1,00		1,00	
Sim	0,94 (0,88; 1,00)	0,044	0,93 (0,90; 0,97)	0,001
Diabetes				
Não	1,00		1,00	
Sim	1,14 (1,11; 1,17)	<0,001	1,05 (1,04; 1,07)	<0,001
Obesidade				
Não	1,00		1,00	
Sim	1,42 (1,38; 1,46)	<0,001	1,06 (1,04; 1,08)	<0,001
**Uso de ventilação mecânica invasiva**				
Não			1,00	
Sim			2,15 (2,11; 2,20)	<0,001
**Admissão em unidade de terapia intensiva**				
Não			1,00	
Sim			1,77 (1,73; 1,81)	<0,001
**Tempo entre o início dos sintomas e a admissão hospitalar** (dias)	1,00 (1,00; 1,00)	0,016	0,98 (0,98; 0,99)	<0,001
**Duração da hospitalização** (dias)	1,01 (1,01; 1,01)	<0,001	1,00 (1,00; 1,00)	<0,001
**Variantes do SARS CoV**-2				
Gama	1,00		1,00	
Delta	0,98 (0,91; 1,05)	0,541	0,82 (0,78; 0,85)	<0,001
Ômicron	0,79 (0,75; 0,84)	<0,001	0,75 (0,73; 0,78)	<0,001
**Vacinação contra a covid**-19				
Não vacinado	1,00		1,00	
Incompleta	0,95 (0,92; 0,99)	0,005	0,99 (0,97; 1,01)	0,201
Primária	0,98 (0,92; 1,05)	0,541	1,00 (0,96; 1,04)	0,917
Completa	0,78 (0,73; 0,85)	<0,001	0,84 (0,81; 0,88)	<0,001

Indivíduos infectados pela variante Ômicron tiveram menor risco de ventilação mecânica (RP 0,79; IC95% 0,75; 0,84) e óbito (RP 0,75; IC95% 0,73; 0,78) comparados àqueles infectados pela variante Gama. A vacinação completa reduziu o risco de ventilação mecânica (RP 0,78; IC95% 0,73; 0,85) e óbito (RP 0,84; IC95% 0,81; 0,88) em relação aos não vacinados. 

O uso de ventilação mecânica foi associado ao óbito (RP 2,15; IC95% 2,11; 2,20), assim como a admissão em UTI (RP 1,77; IC95% 1,73; 1,81).

## Discussão

Este estudo identificou mudanças significativas entre as variantes Gama, Delta e Ômicron em termos de perfil demográfico, manifestações clínicas, comorbidades e desfechos. A variante Ômicron apresentou associação com menor gravidade clínica, refletida em menores taxas de ventilação mecânica invasiva e mortalidade. Observou-se que a vacinação completa ofereceu proteção substancial contra desfechos graves, com impacto mais evidente durante a predominância da Ômicron. Idade avançada, sexo masculino e comorbidades como obesidade foram identificados como importantes fatores de risco para desfechos adversos, o que reforçou a vulnerabilidade desses grupos e a necessidade de estratégias preventivas eficazes, como a vacinação.

A distribuição de pacientes entre as três variantes variou significativamente, com aumento progressivo na idade mediana dos pacientes ao longo dos períodos, sendo a mais alta observada durante o período da Ômicron. Esses achados estão em consonância com os dados obtidos no principal centro médico universitário em Liubliana, Eslovênia, entre 2021 e 2022, onde se observou a predominância de pacientes idosos durante a onda da variante Ômicron (20). Em contrapartida, populações mais jovens também foram identificadas nesse período, em diferentes contextos geográficos e institucionais (16,21). A maior idade mediana entre os pacientes infectados durante a circulação predominante da Ômicron tem sido associada à maior eficácia da vacinação em indivíduos mais jovens (22,23). Além disso, na Eslovênia, observou-se que a menor gravidade da Ômicron possibilitou a hospitalização de pacientes mais idosos e frágeis mesmo diante de quadros menos severos, diferentemente do observado durante a onda da variante Delta, marcada por maior gravidade e demanda hospitalar (20).

Ao analisar os sintomas clínicos entre os três períodos, observou-se menor prevalência de sintomas graves, como dispneia e níveis baixos de saturação de oxigênio, durante a Ômicron. Isso foi consistente com o observado em uma grande rede de saúde comunitária nos Estados Unidos, entre março de 2020 e fevereiro de 2022, na qual foram avaliados 9.582 pacientes hospitalizados com covid-19 durante os períodos pré-Delta, Delta e Ômicron (24). Eles também relataram que a frequência de baixos níveis de oxigênio foi menor em pacientes vacinados durante os períodos da Delta e Ômicron.

A vacinação completa esteve associada à redução do desconforto respiratório, febre e dispneia em pacientes com síndrome do desconforto respiratório agudo relacionada à covid-19, conforme resultados observados na Grécia, entre junho de 2021 e fevereiro de 2022, em uma coorte de 265 pacientes intubados. Durante esse período, a variante Delta foi predominante no país, e pacientes totalmente vacinados apresentaram menor gravidade clínica e menor probabilidade de evolução para óbito (25). Este estudo reforçou que a vacinação completa reduz o risco de mortalidade e a necessidade de ventilação mecânica invasiva, mesmo em pacientes com comorbidades, destacando seu papel na mitigação da gravidade da covid-19.

Neste estudo, a duração da hospitalização no período da Ômicron foi maior (mediana de uma semana) comparada à de achados anteriores que relataram internações mais curtas (26,27). Essa diferença pode ser explicada pela inclusão de casos mais graves de SRAG nesta análise, embora os achados sejam consistentes com o contexto norte-americano mencionado anteriormente (24). Durante a Ômicron, a hospitalização foi significativamente mais curta para pacientes com mais doses de vacina, o que reforçou o efeito cumulativo da vacinação. Na Noruega, entre fevereiro e novembro de 2021, também foram encontradas estadias mais curtas e menor risco de internação em UTI para pacientes vacinados, embora neste estudo não tenha avaliado UTI separadamente (28). A Ômicron parece estar associada à progressão mais rápida dos sintomas do trato respiratório superior, o que pode explicar o tempo reduzido entre o início dos sintomas e a hospitalização, em comparação com a Delta (29,30).

O período da Ômicron apresentou as menores taxas de desfechos graves, como ventilação mecânica invasiva e óbitos, resultados corroborados anteriormente (16,24,26,27,31). Durante a onda da variante Ômicron, observada principalmente entre novembro e dezembro de 2021, diferentes regiões relataram reduções significativas na gravidade clínica da covid-19. Na África do Sul, entre 971 pacientes hospitalizados, registraram-se menores dependência de oxigênio e de ventilação mecânica e mortalidade em comparação com ondas anteriores (26). No sistema de saúde Johns Hopkins, nos Estados Unidos, dados de 2.027 pacientes indicaram menores taxas de internação em UTI, ventilação mecânica e óbito em relação à variante Delta (16). Em Paris, entre 29 de novembro de 2021 e 10 de janeiro de 2022, a análise de 1.716 atendimentos em departamentos de emergência também revelou reduções nas taxas de internação em UTI, uso de ventilação mecânica e mortalidade (27). Entre julho de 2021 e janeiro de 2022, em um hospital acadêmico em Los Angeles, Califórnia, observaram-se igualmente menores taxas de ventilação mecânica e mortalidade durante a predominância da variante Ômicron, em consonância com os achados desta pesquisa (31).

A regressão de Poisson revelou que idade avançada, sexo masculino e raça/cor da pele parda/negra aumentaram a prevalência de ventilação mecânica e óbito, resultados compatíveis com dados obtidos no Brasil entre fevereiro de 2021 e janeiro de 2022, que também identificaram a idade como fator de risco, especialmente entre vacinados hospitalizados (32). Foram analisados 72.647 pacientes internados por covid-19 grave e, entre estes, os homens tiveram maior risco de mortalidade, o que foi consistente com as informações desta pesquisa. A vacinação foi novamente destacada como fator protetor, o que reduziu significativamente o risco de ventilação mecânica e óbito. No Brasil, a administração de doses de reforço vacinal contra a covid-19 aumentou a eficácia na prevenção de casos graves e óbitos, com efeito protetor sustentado por várias semanas após a aplicação (33). Contudo, na Noruega, a partir de dados de pacientes hospitalizados entre fevereiro e novembro de 2021 (28), sugeriu-se que a vacinação pode não alterar substancialmente o risco de mortalidade em pacientes muito idosos, especialmente aqueles com mais de 80 anos.

Esses achados destacam a importância de estratégias de imunização contínuas para mitigar desfechos graves da covid-19, especialmente em populações idosas e de maior risco. A redução progressiva da proteção vacinal ao longo do tempo, atribuída à imunossenescência e à menor resposta imunológica (34,35), torna essencial a aplicação de doses de reforço, que mantêm a eficácia elevada temporariamente (36-38). A introdução de doses de reforço durante a predominância da Ômicron pode ter contribuído para a menor gravidade dos casos observados, evidenciando a necessidade de políticas vacinais atualizadas para prolongar a imunidade e proteger os mais vulneráveis.

Este estudo destacou-se pelo grande tamanho amostral, que conferiu elevado poder estatístico, e pela seleção criteriosa da análise estatística, o que garantiu a robustez dos resultados. Como em qualquer estudo de larga escala, perdas amostrais eram esperadas, mas foram minimizadas por meio de critérios rigorosos de inclusão e exclusão, assegurando a qualidade dos dados e reduzindo potenciais confundidores.

Algumas limitações devem ser consideradas. A identificação das variantes do SARS-CoV-2 foi indireta, baseada na predominância em períodos específicos, o que pode ocasionar imprecisões, especialmente em regiões com menor cobertura genômica. A ausência de dados detalhados sobre tipo de vacina, número de doses e intervalo exato desde a vacinação limitou análises mais específicas sobre esquemas vacinais. A inclusão de apenas pacientes hospitalizados restringiu a generalização dos achados, e fatores como coinfecções, heterogeneidade no atendimento e variações regionais podem ter influenciado os desfechos. Além disso, um possível viés de seleção deve ser considerado, pois a amostra representou apenas uma fração dos casos de SRAG no período analisado.

Embora o grande tamanho amostral tenha fortalecido os achados, pequenas diferenças podem não ter relevância clínica. Por ser um estudo transversal, apenas associações puderam ser identificadas, não relações de causalidade. Estudos longitudinais são necessários para uma avaliação mais aprofundada dos impactos da vacinação e das variantes nos desfechos clínicos da covid-19.

Os achados deste estudo destacaram a dinamicidade da pandemia de covid-19 e a importância de monitoramento contínuo das variantes do SARS-CoV-2 para orientar estratégias de saúde pública. A menor gravidade clínica associada à variante Ômicron, aliada ao impacto positivo da vacinação na redução de desfechos graves, reforçou a necessidade de altas coberturas vacinais e da adaptação das campanhas de imunização diante do surgimento de novas variantes. A identificação de fatores de risco, como idade avançada, sexo masculino e comorbidades, enfatizou a relevância de abordagens preventivas direcionadas a grupos vulneráveis. Esses resultados forneceram evidências fundamentais para a formulação de políticas de mitigação da covid-19, ressaltando o papel essencial da vacinação e da vigilância epidemiológica na contenção dos impactos da pandemia.

## Data Availability

O banco de dados e os códigos de análise utilizados na pesquisa estão disponíveis em: https://github.com/leticiaraposo/Vacinacao_Variantes_covid19_Desfechos_Brasil_2021_2022.

## Material suplementar

Figura suplementar 1.
